# Loss of HES1 Expression is Associated with Extracellular Matrix Remodeling and Tumor Immune Suppression in KRAS Mutant Colon Adenocarcinomas

**DOI:** 10.21203/rs.3.rs-2489562/v1

**Published:** 2023-02-15

**Authors:** Lei Wang, Wenchao Gu, Matthew Kalady, Wei Xin, Lan Zhou

**Affiliations:** Case Western Reserve University; University of Tsukuba; Ohio State University Wexner Medical Center; University of South Alabama Hospital; Case Western Reserve University

**Keywords:** HES1, KRAS, colorectal cancer, extracellular matrix, immune microenvironment

## Abstract

The loss of HES1, a canonical Notch signaling target, may cooperate with KRAS mutations to remodel the extracellular matrix and to suppress the anti-tumor immune response. While HES1 expression is normal in benign hyperplastic polyps and normal colon tissue, HES1 expression is often lost in sessile serrated adenomas/polyps (SSAs/SSPs) and colorectal cancers (CRCs) such as those right-sided CRCs that commonly harbor BRAF or KRAS mutations. To develop a deeper understanding of interaction between KRAS and HES1 in colorectal carcinogenesis, we selected microsatellite stable (MSS) and KRAS mutant or KRAS wild type CRCs that show aberrant expression of HES1 by immunohistochemistry. By comparing the transcriptional landscapes of microsatellite stable (MSS) CRCs with or without nuclear HES1 expression, we investigated differentially expressed genes and activated pathways. We identified pathways and markers in the extracellular matrix and immune microenvironment that are associated with mutations in KRAS. We found that loss of HES1 expression positively correlated with matrix remodeling and epithelial-mesenchymal transition (EMT) but negatively correlated with tumor cell proliferation. Furthermore, loss of HES1 expression in KRAS mutant CRCs correlates with a higher M2 macrophage polarization and activation of IL6 and IL10 immunosuppressive signature. Identifying these HES1-related markers may be useful for prognosis and developing treatment of KRAS-mutant CRCs.

## Background

HES1 (Hairy and enhancer of split 1) is a basic helix-loop-helix transcriptional factor that is expressed in the nuclei of normal intestinal epithelial cells and plays an important role in maintaining intestinal proliferative crypts and regulating enterocyte differentiation (1). HES1 expression is moderated by the Notch pathway, a highly conserved pathway that regulates cellular proliferation and differentiation. In many tumors, aberrant Notch activation can contribute to cancer cell stemness, tumor cell proliferation, metastasis, and the reshaping of the tumor microenvironment (2–5). Notch activation leads to the release of the Notch intracellular domain, which translocates to the nucleus and activates transcription of numerous downstream target genes, including *HES1, HES2, HEY1, HEY2*, and *DTX1*.

The precise role of HES1, a canonical downstream transcription repressor of Notch, in intestinal carcinogenesis is controversial, with studies differing on the relationship between HES1 and colorectal cancer (CRC) outcomes. Although Weng et al. found that high expression of *HES1* mRNA correlated with poor prognosis (6), Ahadi et al. used immunohistochemistry to demonstrate that loss of HES1 expression predicted worse prognoses in CRC patients (7). These contrasting findings may be due to the presence of HES1 in the nuclei of both stromal cells and immune cells in cases where tumor cells are negative for HES1. Thus, studying HES1 in CRC progression using transcriptional expression may result in inconsistent findings. Alternatively, aberrant HES1 signaling may have distinct roles in different CRC pathways and in tumors with different genetic background.

HES1 may be related to CRC progression initiated by *KRAS* or *BRAF* mutations. In the canonical pathway of colorectal carcinogenesis, loss of the tumor suppressor *APC (adenomatous polyposis coli)* is followed by tumorigenic alterations of TP53, MAPK, and TGF-β signaling (8). Therefore, the great majority of human CRCs, including hereditary syndromes and sporadic cancers, display *APC* mutations. However, CRC progression can be alternatively initiated by *KRAS* or *BRAF* mutations from adenomas with serrated morphologies (9, 10), and previous results suggest a relationship between HES1 and these pathways. Differentiation and proliferation of intestinal epithelium mediated by mutant *KRAS* was linked to activation of HES1, in a mouse model and human HP (11). We reported that, on the contrary, loss of HES1 expression is observed in the majority of sessile serrated lesions (SSL) but not in hyperplastic polyps (HP) (12). Moreover, we found that loss of HES1 expression is frequently observed in right-sided colon adenocarcinomas (13), which commonly harbor *KRAS* or *BRAF* mutations (14). Although most of the SSLs and the right-sided CRC with CpG island methylator phenotype (CIMP) arise from *BRAF* mutations, *KRAS* mutation is common in both CIMP-negative CRCs and CIMP-high but microsatellite stable (MSS) CRCs (15).

To gain insight into the regulation between KRAS and HES1 in colorectal carcinogenesis, we examined microsatellite stable (MSS) and *KRAS* mutant CRCs that show aberrant expression of HES1 by immunohistochemistry. Using RNA sequencing and Nanostring’s RNA array analysis, we investigated differentially expressed genes and activated pathways regulated by *HES1* in *KRAS* mutant CRCs.

## Materials And Methods

### Patients

The study of archived human CRC was approved by the Institutional Research Board (IRB) of the University Hospitals Case Medical Center, Cleveland Clinic Foundation, and Fudan University Shanghai Cancer Center. Informed consent was obtained from patients who agreed to donate tissues for the purpose of research according to the regulation by the IRB. All cases included in the study were confirmed as colorectal adenocarcinoma by two experienced pathologists. All methods used in this study were carried out in accordance with IRB guidelines and regulations. Demographic and clinicopathological data were collected from the medical records. Mutational status was determined by the ColonCore next-generation sequencing (NGS) panel (Burning Rock Biotech, Guangzhou, China) which is designed for simultaneous detection of microsatellite instability (MSI) status and mutations in 38 CRC related genes. Cases included in this study were microsatellite stable (MSS) CRCs that also had *KRAS* and *APC* mutation (Table 1). Another cohort of MSS and *KRAS* wild type CRCs were included. These cases were part of an IRB-approved annotated biobank. Biobank tumors had been previously evaluated for microsatellite instability and *KRAS* mutation status as previously described (15). Cases that carry other frequently mutated genes (*TP53, BRAF, NRAS*) were excluded from the study.

### Gene expression analysis

Ten cases, 5 HES1 (−) and 5 HES1 (+), were subjected to RNA sequencing (cohort 1). RNA of these cases was isolated from the fresh frozen tissue followed by mRNA library preparation using Illumina’s TruSeq RNA Sample Prep Kit v2 (Illumina, RS-122–2001, San Diego, CA, USA). Sequencing was performed using Illumina HiSeq 2500 System (Illumina, San Diego, CA, USA). Another cohort of 9 cases, 4 HES1 (−) and 5 HES1 (+) were subjected to Nanostring RNA gene expression array analysis (cohort 2). RNA was isolated from formalin-fixed, paraffin-embedded (FFPE) tissue and assessed by the nCounter^®^ PanCancer IO 360^™^ Panel (NanoString technologies, Seattle, WA, USA) (16).

### TCGA data acquisition

TCGA data of colorectal cancer (n=431) level 3 gene-expression (counts) and somatic mutation were obtained from Genomic Data Commons (GDC) (https://portal.gdc.cancer.gov/). *KRAS* mutation was identified based on the “maftool” R package. The original counts data were transformed into transcript per kilobase million (TPM). Patients who lacked follow-up and somatic mutation information were excluded. A total of 155 colorectal cancer patients with *KRAS* mutation and 222 colorectal cancer patients with wild type *KRAS* were enrolled in this study. The high and low expression of HES1 in TCGA data was determined by the median expression (10.799) as a cutoff. Survival analysis was performed with Kaplan-Meier analysis in all colorectal cancer patients.

### Differentially expressed gene (DEG) and GSEA analysis

To identify genes associated with HES1 expression, DEGs was determined by using limma R package. The significant criteria were selected using P value < 0.05 and absolute fold-change (FC) >1. The Venn Diagram was generated by the package of “venn”. Gene Set Enrichment Analysis (GSEA) analysis was performed by “ClusterProfiler” package.

### Immune cell infiltration analysis

Immune cells signature was determined by previously published method (17). Briefly, Gene Set Variation Analysis (GSVA) was used to calculate the scale of value of each immune cells.

### Immunohistochemical staining and evaluation

Paraffin blocks of 25 cases from cohort 1 & 2 were selected for the construction of the tissue microarray (TMA). For each block, three cores with a diameter of 2 mm were obtained from the tumor. Immunohistochemical staining (IHC) was performed using the automated immunostainer (Ventana, Tucson, AZ, USA). Primary antibodies used in this study include HES1 (Clone: EPR4226, Cat. No. ab108937, Abcam), Ki67 (Clone: 30–9, Cat. No. 790–4286, Ventana), TP53 (Clone: DO-7, Cat. No. M7001, Dako), RB1 (Clone: 4H1, Cat. No. 9309, Cell Signaling Technology), Cyclin D1 (Clone: SP4-R, Cat. No. 790–4508, Ventana), E-cadherin (Clone: NCH-38, Cat. No. M3612, Dako), Vimentin (Clone: V9, Cat. No. IR630, Dako), CD44 (Clone: DF1485, Cat. No. M7082, Dako), CD8 (Clone: SP57, Cat. No. 790–4460, Ventana), CD163 (Clone: MX081, Cat. No. MAB-0869, Fuzhou Maixin Biotechnology), CD68 (Clone: KP1, Cat. No. M-0160–1.0, Shanghai Changdao Biotechnology), phospho-STAT3 (Tyr705, Clone: D3A7, Cat. No. 9145, Cell Signaling Technology), IL10 (Clone: 2472A, Cat. No. MAB91842, R&D Systems). Expression of HES1 was evaluated as previously described (12). The presence of HES1 nuclear expression was considered HES1 (+), while loss of HES1 nuclear expression was classified as HES1 (−). Histoscores (H-scores) were calculated by multiplying the staining intensity (0=negative, 1=weak, 2=moderate, 3=strong) and the percentage of positive cells (number of positive tumor cells/ number of total tumor cells, range 0–100). All cases were scored by two experienced pathologists. The expression status of TP53, RB1 and Cyclin D1, IL10 were evaluated using H-scores. Tumor cells showed homogeneously strong membrane expression of E-cadherin were considered positive, while weak or loss expression of E-cadherin of tumor cells was classified as abnormal. The percentage of positive Ki67 staining in tumor cells was evaluated. Densities of CD8, CD163 and CD68 were calculated (area of positive immune cells/total area of tissue).

### Statistical Analysis

Data were analyzed using R software (version4.2.1). Comparisons of ≥ 2 groups were conducted using a parametric test (Student t-test or ANOVA test) or a nonparametric test (Wilcoxon rank-sum test or Kruskal-Wallis test, Pearson Chi-Square test or Fisher’s Exact Test). ns, *, **, and *** represent not significant (p ≥ 0.05), p < 0.05, p ≤ 0.01, and p ≤ 0.001, respectively.

## Results

### Identification of biological pathways correlated with HES1-loss in *KRAS* mutant CRCs

**Figure 1:**
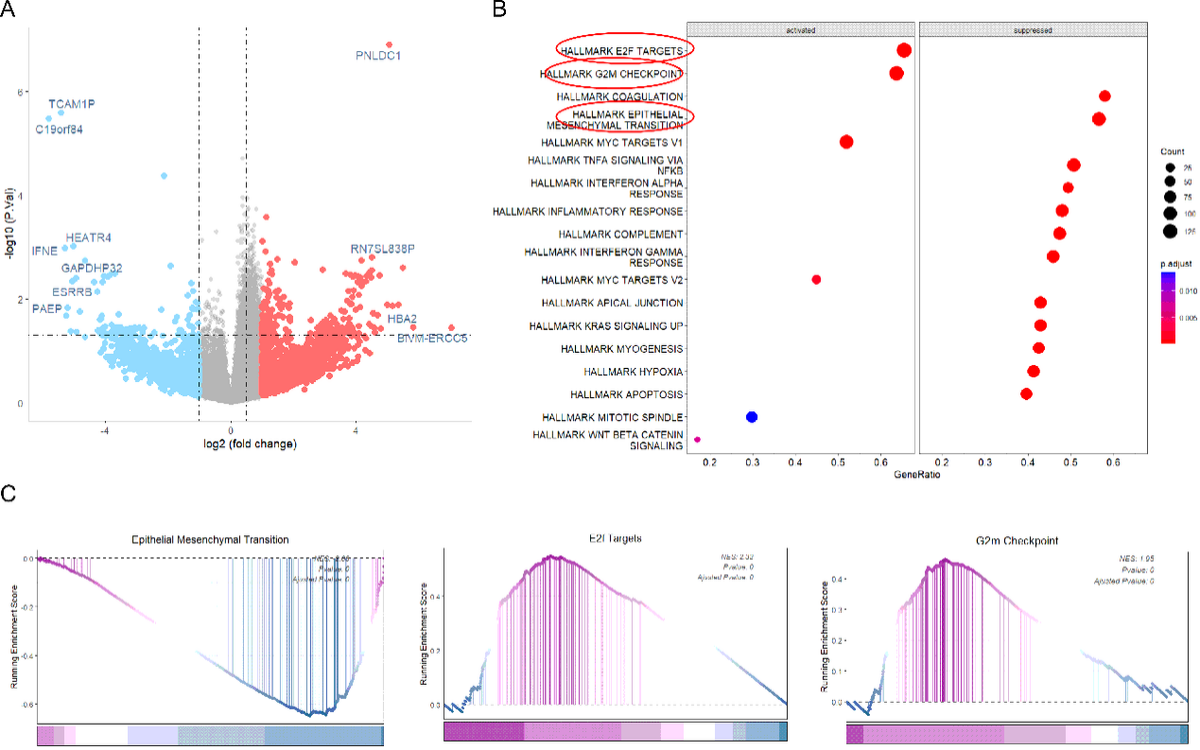
RNA sequencing analyses. (A) A total of 360 DEGs were obtained between HES1 (+) group (n=5) and HES1 (−) group (n=5) (p<0.05), of which 248 DEGs were downregulated and 112 DEGs were upregulated in HES1 (−) group. (B) The biological pathways implicated by the aberrant HES1 expression revealed by GSEA analysis of the DEGs of HES1 (+) and HES1 (−) groups. (C) GSEA plots showing that HES1 loss was positively correlated with “EPITHELIAL_MESENCHYMAL_TRANSITION” signaling, but negatively correlated with “E2F_TARGETS” and “G2M_CHECKPOINT” signaling.

We found that loss of HES1 nuclear expression is more frequently associated with CRCs harboring *BRAF* or *RAS* mutations (13) (14). To understand the reciprocal regulation between KRAS and HES1, we examined the expression of HES1 in *KRAS* mutant CRCs. These *KRAS* mutant cases (Table 1) were sequenced by the ColonCore next-generation sequencing panel. We selected 5 cases with HES1 nuclear expression, referred as HES1 (+), and 5 cases with loss of HES1 nuclear expression, referred as HES1 (−) (Fig S1), in cohort 1. All cases had *KRAS* and *APC* mutation while other frequently mutated genes were wild type (Table 1S). In addition, all ten cases were determined to be MSS. RNA sequencing of this cohort revealed 360 differentially expressed genes (DEGs), of which 248 were significantly downregulated while 112 were upregulated in the HES1 (−) group (Fig 1A). To investigate the biological pathways implicated by aberrant HES1 expression, we subjected these DEGs to GSEA. We found that HES1-loss positively correlated with EPITHELIAL_MESENCHYMAL _TRANSITION but negatively correlated with E2F_TARGETS and G2M_CHECKPOINT (Fig 1B &1C).

**Figure 2:**
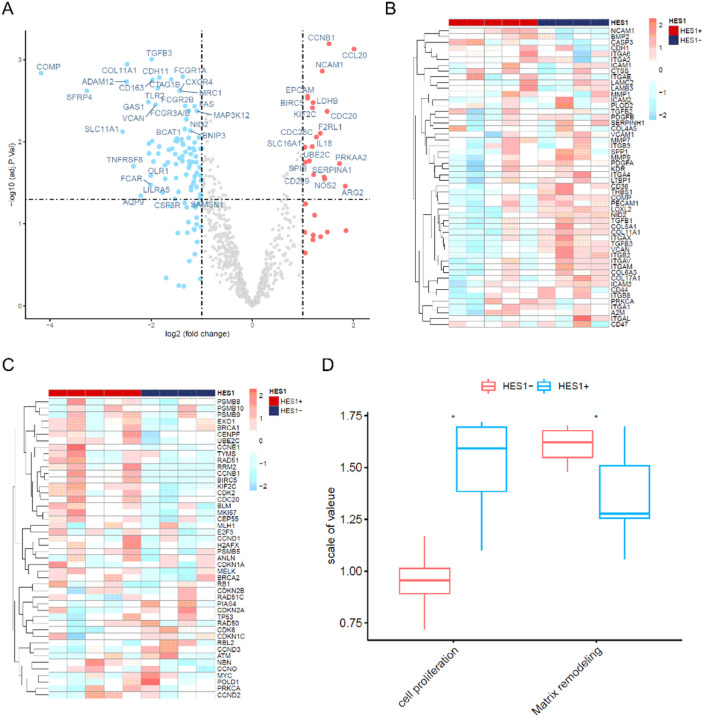
Nanostring RNA gene expression array analyses. (A) Volcano plot identified 93 DEGs between HES1 (+) (n=5) and HES1 (−) group (n=4) (p<0.05), of which 82 DEGs were upregulated and 19 DEGs were downregulated in HES1 (−) group. (B) A heatmap of 52 matrix remodeling and metastasis process-related genes (rows) is shown for 9 samples (columns) including HES1 (−) (blue bar) and HES1 (+) cases (red bar). (C) A heatmap of 46 differentially expressed genes included in the cell proliferation process (rows) for 9 samples (columns) including HES1 (−) (blue bar) and HES1 (+) cases (red bar). (D) The GSVA plot showing that loss of HES1 was positively correlated with the matrix remodeling and metastasis process and negatively correlated with cell proliferation process (* p<0.05).

To verify these findings, we selected another cohort of nine cases (cohort 2) including 5 HES1 (+) and 4 HES1 (−). We examined the transcriptional profile of these cases using the NanoString nCounter PanCancer IO 360 panel, which profiles 750 cancer-related human genes across 16 key immuno-oncology pathways both within the tumors and at the interface of tumor stroma interaction and tumor immune responses. All these cases were MSS and had mutations in *KRAS* and *APC*. Other frequently mutated genes were wild type (Table 2S). Of the 93 DEGs found, 19 were downregulated and 82 upregulated in the HES1 (−) group compared to the HES1 (+) group (Fig 2A). Consistent with RNA sequencing analysis in cohort 1, HES1-loss positively correlated with matrix remodeling and metastasis (Fig 2B) and negatively correlated with cell proliferation (Fig 2C & 2D).

To assess if these differentially regulated signaling pathways are uniquely associated with *KRAS* mutation, we selected a cohort of *KRAS* wild type (WT) CRC cases (cohort 3), composed of six HES1 (+) and six HES1 (−) samples. Analysis of the transcriptional profile using the NanoString nCounter PanCancer IO 360 panel identified 12 DEGs between the HES1 (+) (n=6) and the HES1 (−) group (n=6), of which 6 DEGs were upregulated and 6 downregulated in the HES1 (−) group. Among these, *LAMA1* (Laminin Subunit Alpha 1), which encodes the extracellular matrix glycoprotein,displayed a higher expression in HES1 (−) groupthan in HES1 (+) group.However, unlike *KRAS* mutant CRCs, matrix remodeling and cell proliferation were not associated with HES1-loss in *KRAS* WT CRCs (Fig S2).

### Differential EMT marker and proliferation marker expression is regulated by HES1 in *KRAS* mutant CRCs

**Figure 3:**
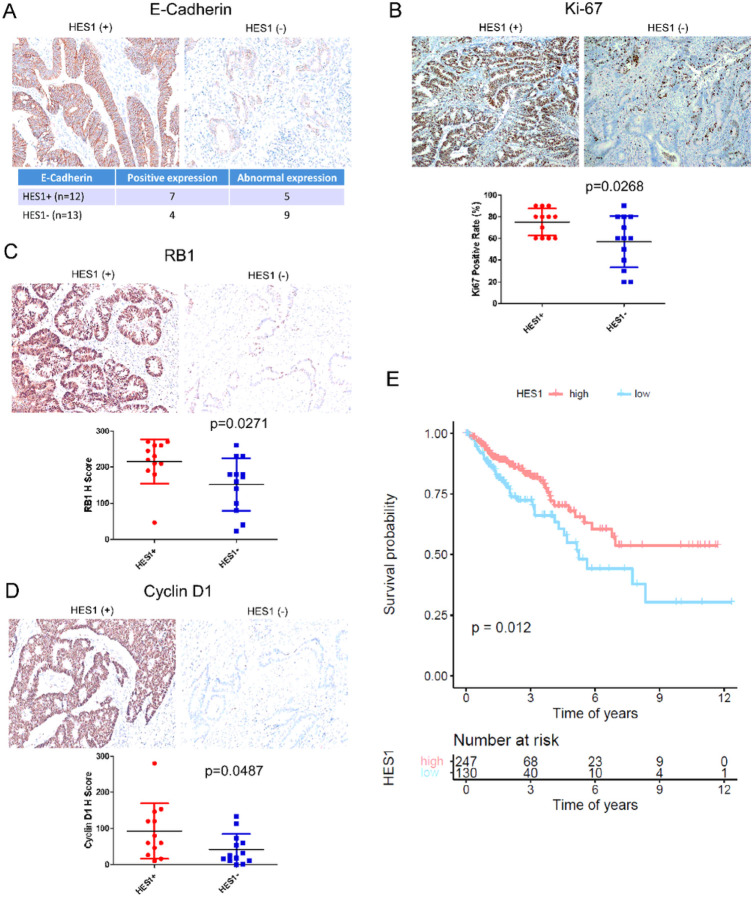
Expression of EMT and proliferation markers. (A) Representative immunohistochemistry staining of E-cadherin and the analysis of its aberrant expression associated with HES1 (−) CRCs. (B-D) Representative immunohistochemistry staining of Ki67 (B), RB1 (C) and Cyclin D1 (D). Differential IHC scores were shown below. (E) Poor prognosis associated with low HES1 expression in TCGA CRC dataset.

The results from the RNA sequencing and the NanoString analysis showed consistent positive correlation between HES1-loss and tumor migration and invasion but negative correlation with tumor proliferation. We assessed the expression of EMT markers such as E-cadherin, Vimentin and CD44, finding that HES1 (−) CRCs more frequently show weak or negative staining of E-cadherin compared to HES1 (+) CRCs. Weak expression or no expression of E-cadherin is observed in 69.2% (9/13) of the HES1 (−) group but in 41.7% (5/12) of the HES1 (+) group (Fig 3A). There is no significant difference in the expression of Vimentin or CD44 between these two groups (data not shown). However, three HES1 (−) cases exhibited liver metastases identified upon CRC diagnosis (Table 1), suggesting a more rapid progression of CRCs compared to HES1 (+) cases, where no liver metastases were found.

We also assessed the expression of the cell cycle related markers (Ki67, TP53, RB1 and Cyclin D1). Compared to HES1 (−) tumors, expression levels of Ki67 (p=0.0268) (Fig 3B), RB1 (p=0.0271) (Fig 3C) and Cyclin D1 (p=0.0487) (Fig 3D) were all significantly upregulated in the HES1 (+) tumor cells. TP53 (p=0.2664) had a trend of increased expression in the HES1 (+) group (data not shown). Corroborating RNA sequencing and transcriptional profiling by RNA array, analysis of TCGA CRC data set identified a worse prognosis in patients who have lower expression of HES1 (Fig 3E).

### HES1-loss correlates with higher M2 macrophage signature in *KRAS* mutant CRCs

**Figure 4:**
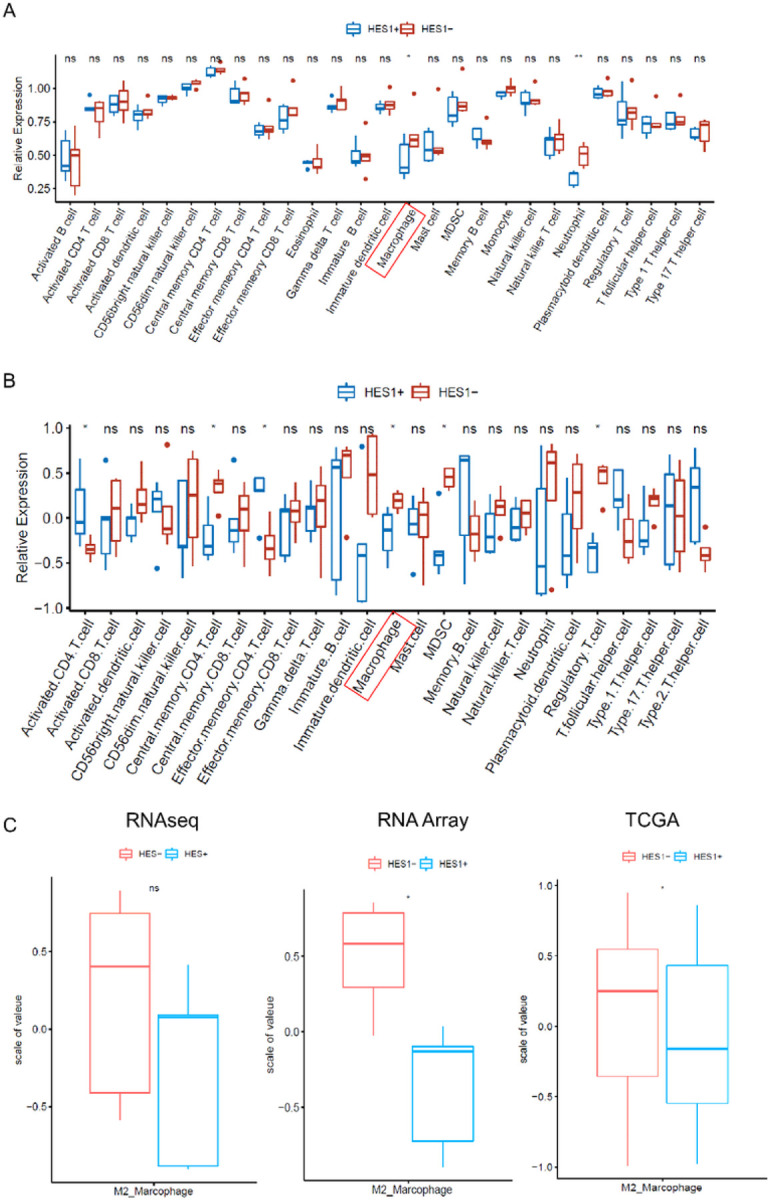
Correlation between HES1 expression and tumor infiltrating immune cell signatures. (A-B) Tumor infiltrating immune cell signature was analyzed. The signature of macrophage was elevated in HES1 (−) group from both RNA sequencing (A) and Nanostring Array (B) (p<0.05). M2 macrophage were sub-clustered by the expression of CD206, CD204 and CD163. Higher M2 macrophages in HES1 (−) group were observed in RNA sequencing, Nanostring Array and TCGA data (* p<0.05) (C).

A significant set of genes enriched by HES1 (−) CRCs are related to inflammatory pathways and responses, including TNFα signaling via NFKB, the inflammatory response, the interferon α response, and the interferon γ response (Fig 1B). Thus, we evaluated tumor infiltrating immune cells in HES1 (+) and HES1 (−) groups. Higher density of macrophage infiltration in HES1 (−) group was found by both RNA sequencing and NanoString Array (Fig 4A and 4B). In the HES1 (−) group, RNA sequencing found higher neutrophil density in the HES1 (−) group, while NanoString Array analysis found upregulation of regulatory CD4 T cells, myeloid-derived suppressive cells (MDSCs), and regulatory T cells. M2 macrophages were sub-clustered according to the expression of genes including CD206, CD204, and CD163 (18). Both RNA sequencing and the NanoString array found that genes expressed by M2 macrophages were higher in the HES1 (−) group than in the HES1 (+) group. Analysis of M2 macrophage gene expression in the TCGA data set showed a similar increase of M2 macrophage gene expression in patients who have lower expression of HES1 (Fig 4C).

**Figure 5:**
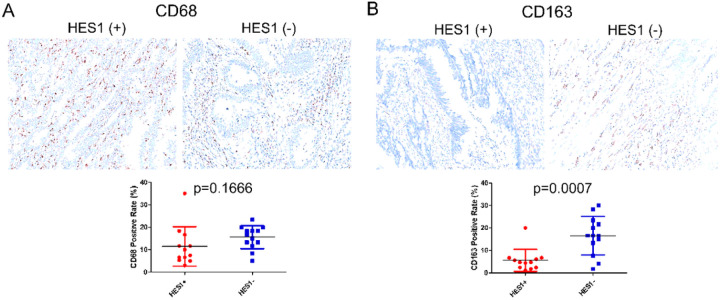
Expression of macrophage markers in HES1 (+) and HES1 (−) CRCs. A pan-macrophage or M1 macrophage marker, CD68 (A), and the M2 macrophage marker, CD163 (B) was examined by IHC, both of which showed cytoplasmic staining. There was no significant difference in the density of CD68 positive macrophages between HES1 (+) and HES1 (−) groups (p=0.1666) (A). The density of CD163 positive macrophages was much higher in HES1 (−) group (p=0.0007) (B).

We confirmed the results from RNA sequencing and NanoString array with IHC. CD68-positive or CD163-positive macrophages were mainly detected in the tumor stroma. While CD68 is normally considered a pan-macrophage or M1 macrophage marker, CD163 is accepted as a M2 macrophage marker (19, 20). We found no obvious difference in the density of CD68-expressing macrophage between the HES1 (+) and HES1 (−) groups (p=0.179) (Fig 5A). However, the density of CD163-positive macrophage was much higher in the HES1 (−) group than in the HES1 (+) group (p=0.0007) (Fig 5B) confirming transcriptome profiling analysis.

### Signaling pathways in IL6 and IL10 correlates with higher M2 macrophage in HES1-loss *KRAS* mutant CRCs

**Figure 6:**
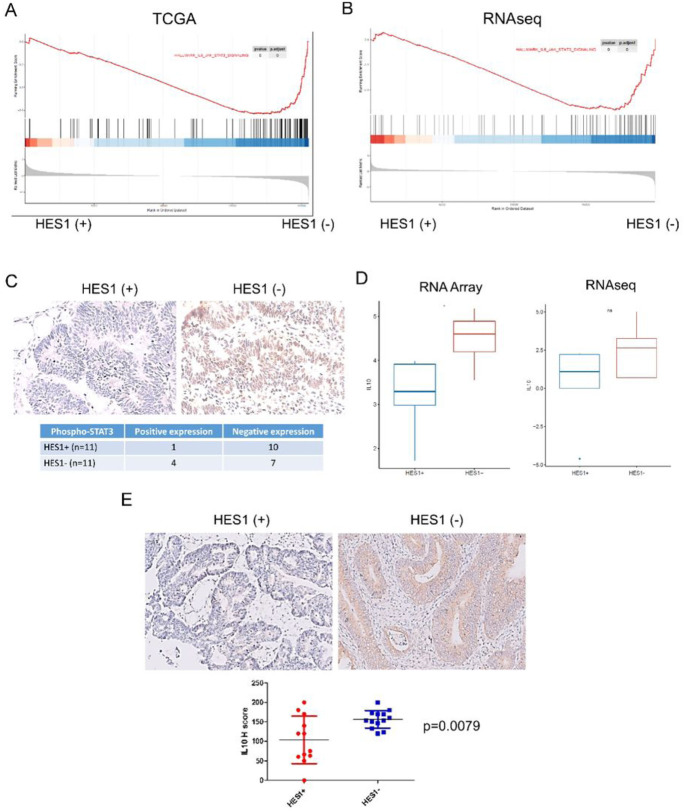
Signaling pathways involved in higher M2 macrophage in HES1-loss *KRAS* mutant CRC. (A-B) “IL6_JAK_STAT3” signaling activation in HES1(−) group was identified by GSEA analysis of TCGA data set(A) and RNA sequencing (B). (C) The expression of phospho-STAT3 was evaluated by IHC, which displayed nuclear staining. Numbers of phospho-STAT3 (+) cases was higher in HES1 (−) group than in HES1 (+) group. (D-E) Expression of M2 macrophage related cytokine, IL10, was assessed by RNA array (* p<0.05) and RNA sequencing (D) as well as by IHC (E).

To explore the significance of inflammatory response and tumor-associated macrophage (TAM) linked to HES1-loss in *KRAS* mutant CRC in a larger database, we performed GSEA analysis of HES1-high and HES1-low *KRAS* mutant CRC cases in the TCGA dataset. GSEA analysis found that HES1-low positively correlated with IL6_JAK_STAT3_SIGNALING (Fig 6A). A similar signature was observed from RNA sequencing analysis (Fig 6B). IL6 secreted by TAMs promotes CRC proliferation and invasion through IL6/STAT3 signaling (21). Accordingly, IHC staining showed that tumor cells in 36.4% (4/11) cases of HES1 (−) group were highly positive for phospho-STAT3 while only 9.1% (1/11) of HES1 (+) cases were positive for phospho-STAT3 (p=0.311) (Fig 6C).

We then examined other cytokines associated with HES1-loss that may potentiate M2 macrophage accumulation. NanoString Array analysis found that IL10 mRNA expression was upregulated in the HES1 (−) group. RNA sequencing also revealed a positive trend of IL10 expression in the HES1 (−) group (Fig 6D). Using IHC, we found that IL10, which polarizes macrophages towards the M2 phenotype (22), shows expression mainly by tumor cells. Overall, IL10 expression in the HES1 (−) group was higher than in the HES1(+) group (p=0.0079) (Fig 6E). Our results thus suggest that IL10 released from HES1 (−) CRC tumors into the TME may play a role in M2 macrophage polarization.

## Discussion

The role of Notch signaling in colorectal carcinogenesis and progression remains controversial. Reports have shown that Notch activation and Wnt signaling act synergistically to promote the initiation of adenoma formation (23). Notch activation by copy number gain of *NOTCH1* is associated with a worse clinical prognosis (24). Activated Notch signaling combined with additional oncogenic driver mutations also drive CRC invasion and metastasis in animal models (25, 26). Further, reports have shown that Notch signaling activation and *KRAS* mutation is significantly associated with poor prognosis in human CRC (25). On the contrary, our group found that nuclear HES1 expression is lost in 91% of sessile serrated adenomas/polyps (SSA/p) and most of the right-sided colorectal cancer which commonly harbors *BRAF* or *KRAS* mutation (12) (13). It has been proposed that KRAS mutation creates a subset of CRCs that arises *via* the serrated pathway (27). However, the mechanisms of aberrant Notch signaling in the progression of human CRC and its cross-regulation with KRAS are still unclear.

In this study, we focused on the MSS CRCs that carry *KRAS* mutation to assess how aberrant HES1 expression impacts genes and pathways that may affect CRC tumorigenesis and progression. Because most of the *KRAS* mutant CRCs also have *APC* mutation, we included both in our study cohorts and group these cases into HES1 (+) and HES1 (−) groups according to the presence or absence of tumor nuclear expression of HES1. By using two different transcriptome profiling approaches, we identified commonly affected pathways regulated by HES1, a canonical target of Notch signaling.

Our work revealed that loss of HES1 expression positively correlated with matrix remodeling and epithelial-mesenchymal transition (EMT) but negatively correlated with tumor cell proliferation. These findings indicated that absence of nuclear HES1 expression suppresses tumor cell proliferation but also promotes CRC invasion. Uncontrolled proliferation and invasion are the dominant characteristics of malignant tumors (28). However, these two cellular processes do not always occur simultaneously, a phenomenon described as migration-proliferation dichotomy (29, 30). Indeed, we found that loss of HES1 correlated with decreased E-cadherin expression in CRC. Decreased expression of E-cadherin on epithelial cell surface is a crucial marker of EMT process. Consistently, we found liver metastases in 25% of patients whose colon lesions lost HES1 expression when diagnosed. On the other hand, loss of nuclei HES1 CRC cases showed decreased expression of cell cycling markers including Ki67, Cyclin D1, RB1 and TP53. Therefore, HES1 likely functions as a migration-proliferation dichotomy node that controls tumor invasion and proliferation in *KRAS* mutant CRC, and its loss may be a predictor of tumor invasion and metastasis.

Interestingly, we did not find the correlation between HES1 expression and EMT process and cell proliferation in *KRAS* WT CRCs. A correlation with *KRAS* mutant CRC suggests that aberrant HES1 expression may interact with RAS signaling to promote invasion and metastasis. Few studies have focused on the relationship between HES1 expression and *KRAS* mutations. Feng et al. found that, in a mouse model, mutant Kras mediated colon epithelium differentiation and proliferation was linked to activation of Hes1 (11). Kim et al. investigated the clinical significance of HES1 expression in human small intestinal adenocarcinomas (25). Consistent with our findings, patients with *KRAS* mutant tumors that showed loss of HES1 expression had worse prognoses. This study also reported independence between the prognosis of patients with positive HES1 expression and *KRAS* mutation status. We recently found that loss of HES1 expression in CRC was associated with *KRAS* or *BRAF* mutation while almost all the *KRAS/BRAF* mutant tumors located on the right colon show negative HES1 expression (14). However, the exact mechanism linking aberrant HES1 expression to *KRAS/BRAF* mutant tumor invasion and metastasis remains unknown.

Our results also suggest that alteration of HES1 expression in tumor cells can rewire the tumor microenvironment and affect tumor progression through M2 macrophage polarization. Macrophages play a crucial role in tumor immune microenvironment. While M1-like macrophages are commonly referred to as pro-inflammatory and anti-tumoral, M2 macrophages, marked by CD163 and CD206, often present anti-inflammatory and immunosuppressive activities (31). Tumor-associated macrophages (TAMs) in advanced tumors often closely resemble the M2-like macrophages and can exert immunosuppression in the tumor microenvironment (32). TAMs of CRC, polarized to M2-like phenotype by cytokines such as IL4, IL13, IL10, produce anti-inflammatory cytokines including TGFβ and IL10 and are associated with a poor prognosis (33, 34). Consistently, we found that HES1 (−) CRCs had higher density of CD163-positive macrophages and displayed higher level of IL10 when compared to HES1 (+) tumors. Factors produced by the immune cells, stromal cells, and cancer cells regulate all aspects of tumor pathogenesis and progression. IL6, for example, may function as a critical link between inflammation and CRC development (35). Other studies have shown IL6 polarizes M2 macrophage in CRC (36) and that IL6/STAT3 can form a positive feedback loop to stimulate tumor growth and progression (37). The exact mechanism and impact of enhanced IL6/STAT3 signaling in HES1 (−) CRC warrants further investigation. Nevertheless, these findings support a vital role of M2 macrophage polarization and a role of IL10 and IL6/STAT3 signaling in HES1 (−) *KRAS* mutant CRCs that may act in concert to promote tumor progression and metastasis. Finally, RNA array revealed upregulation of MDSCs and regulatory T cells in HES1 (−) CRCs, suggesting these immune cells may also promote HES1 (−) CRC tumorigenesis.

*KRAS* mutations are associated with decreased response rate to anti-EGFR therapy (38, 39). Inhibitors that selectively target KRASG12C is promising but this type of inhibitors has limited mutation targets (40). Most CRC patients, except those whose tumors have high levels of MSI or are deficient in mismatch repair, cannot benefit from FDA-approved immune check point inhibitors (41, 42). Our findings identify signaling pathways and cytokines impacted by HES1 that are responsible for promoting tumor progression in *KRAS* mutant CRCs. These markers may be useful for prognostic prediction and future design of novel therapeutics for *KRAS* mutant CRCs.

## Conclusion

In summary, our study indicates that aberrant HES1 expression correlates with tumor matrix remodeling in *KRAS* mutant CRC. Loss of HES1 also plays a role in rewiring the tumor immune microenvironment to induce immune suppression.

## Figures and Tables

**Table 1.** Patient Demographics and Tumor Characteristics of *KRAS* Mutant Cohorts

		HES1 (+) (n=12)	HES1 (−) (n=14)	*P* value
Age	≥ 60y	7 (58.3%)	10 (71.4%)	0.683
	< 60y	5 (41.7%)	4 (28.6%)	
Sex	Male	7 (58.3%)	9 (64.3%)	1.000
	Female	5 (41.7%)	5 (35.7%)	
Size	≥ 5cm	4 (33.3%)	8 (57.1%)	0.267
	< 5cm	8 (66.7%)	6 (42.9%)	
Location	Left	8 (66.7%)	10 (71.4%)	1.000
	Right	4 (33.3%)	4 (28.6%)	
Differentiation	Low grade	8 (66.7%)	11 (78.6%)	0.665
	High grade	4 (33.3%)	3 (21.4%)	
Infiltration depth	T1/T2	2 (16.7%)	2 (14.3%)	1.000
	T3/T4	10 (83.3%)	12 (85.7%)	
Lymph nodes	Positive	4 (33.3%)	7 (50.0%)	0.453
	Negative	8 (66.7%)	7 (50.0%)	
Distant metastasis	Positive	2 (16.7%)	6 (42.9%; 3 synchronous liver metastases)	0.216
	Negative	10 (83.3%)	8 (57.1%)	
KRAS	G12A	4 (33.3%)	4 (28.6%)	
	G12C	0 (0.0%)	2 (14.3%)	
	G12S	0 (0.0%)	1 (7.1%)	
	G12V	3 (25.0%)	4 (28.6%)	
	G13A	4 (33.3%)	2 (14.3%)	
	G61A	1 (8.3%)	0 (0.0%)	
	G61H	0 (0.0%)	1 (7.1%)	
APC	Frameshift mutation	10 (83.3%)	7 (50.0%)	
	Nonsense mutation	5 (41.7%)	11 (78.6%)	

## Data Availability

The datasets generated and/or analysed during the current study are available in the Genome Sequence Archive (Genomics, Proteomics & Bioinformatics 2021) in National Genomics Data Center (Nucleic Acids Res 2022), China National Center for Bioinformation / Beijing Institute of Genomics, Chinese Academy of Sciences (GSA-Human: HRA003857) that are publicly accessible [https://bigd.big.ac.cn/gsa-human/browse/HRA003857].

## Availability of data and material:

Data and material are available upon request.
